# Response to Sodium Channel blocking Antiseizure medications and coding polymorphisms of Sodium Channel genes in Taiwanese epilepsy patients

**DOI:** 10.1186/s12883-021-02395-2

**Published:** 2021-09-23

**Authors:** Chih-Hsiang Lin, Chen-Jui Ho, Yan-Ting Lu, Meng-Han Tsai

**Affiliations:** 1 Department of Neurology, Kaohsiung Chang Gung Memorial Hospital, Colleague of Medicine, Chang Gung University, Kaohsiung Kaohsiung City, 83301 Taiwan; 2 School of Medicine, College of Medicine, Chang Gung University, Taoyuan, Taiwan

**Keywords:** Antiseizure medications, Sodium channel gene, Single-nucleotide polymorphisms, Drug resistance epilepsy, *SCN1B*

## Abstract

**Background:**

Many antiseizure medications (ASMs) control seizures by blocking voltage-dependent sodium channels. Polymorphisms of sodium channel genes may affect the response to ASMs due to altering the effect of ASMs on blocking sodium channels.

**Methods:**

We conducted a retrospective study of epilepsy patients followed up at the Neurological Department of Kaohsiung Chang Gung Memorial Hospital, Taiwan between January 2010 and December 2018. We categorized the patients into response, partial response, and failure to sodium channel blocking ASM groups. Sodium channel blocking ASMs included phenytoin, carbamazepine, lamotrigine, oxcarbazepine, lacosamide, zonisamide, topiramate, and valproic acid. A subgroup of predominant sodium channel blocking ASMs included phenytoin, carbamazepine, lamotrigine, oxcarbazepine, and lacosamide. Associations between the response of ASMs and single-nucleotide polymorphisms of *SCN1A*, *SCN1B*, *SCN2A*, and *SCN9A* were analyzed.

**Results:**

Two hundred Taiwanese patients and 21 single-nucleotide polymorphisms among *SCN1A*, *SCN1B*, *SCN2A*, and *SCN9A* were evaluated. We found allele C of rs55742440 in *SCN1B* was statistically significantly associated with not achieving seizure-free with sodium channel blocking ASMs. For the predominant sodium channel blocking ASMs group, no SNPs were associated with the response of ASMs.

**Conclusion:**

Single-nucleotide polymorphism in *SCN1B* was associated with the response to sodium channel blocking ASMs. This highlights the possibility that beta subunits may affect the function of sodium channels and resulted in different responsiveness to ASMs.

**Supplementary Information:**

The online version contains supplementary material available at 10.1186/s12883-021-02395-2.

## Background

Epilepsy is a chronic disorder that requires the long-term use of antiseizure medications (ASMs), the choice of ASM is based on the seizure type and epileptic syndrome, and there are currently no reliable biomarkers to predict the responsiveness to ASMs [1]. With the current advance in the development of ASMs, one-third of patients still have seizures despite multiple ASM treatments [2]. According to the International League Against Epilepsy, drug-resistant epilepsy is defined as the failure of adequate trials of two tolerated, appropriately chosen and used ASM schedules (whether as monotherapies or in combination) to achieve sustained seizure freedom [3]. Several theories tried to explain the cause of drug-resistant epilepsy, including the transporter hypothesis [4], the neuronal network hypothesis [5], the intrinsic severity hypothesis [6], the target hypothesis [7], and the gene variant hypothesis [8].

Among these theories, we focused on the gene variant hypothesis, which is the pharmacogenetic association of the responsiveness of ASM and the genetic variant of ASM targets. Sodium channel (SCN) is responsible for the generation and propagation of action potential in neurons, thus many ASMs act by reducing the high-frequency firing of the voltage-dependent SCN that occurred during the seizure [9]. SCN is formed by one alpha subunit and two beta subunits. Alpha subunit functions as the voltage sensor and forms the pore region of the channel, while beta subunits regulate or assist the function of SCN [10]. The alpha subunits are encoded by the *SCN(1–10)A* genes and beta subunits by *SCN(1–4)B* genes. Many studies had investigated the relationship between SCN gene single-nucleotide polymorphisms (SNPs) and drug-resistant epilepsy. Tate et al. reported that the *SCN1A* rs3812718 variant was associated with the maximum dose of phenytoin and carbamazepine for controlling seizures [11]. This SNP has also been reported to be related to drug-resistant epilepsy in different ethnic groups [12–14] but with conflicting results [15–17]. Other SNPs of SCN genes have also been evaluated, but not as extensively [18–20]. A recent study evaluated 39 polymorphisms in the *SCN1A*, *SCN2A*, and *SCN3A* genes in patients from Malaysia and Hong Kong, had found no associations between gene polymorphisms and responsiveness to ASMs [21].

The above mentioned studies used a selective approach by focusing on genotyping a dozen “common” SNPs, many of which were located in introns or non-coding regions (such as UTR or inter-gene areas), and therefore the interpretation of the functional consequence remains unclear. In this study, we adopted a different approach by using next-generation sequencing-based techniques to cover all coding regions of four sodium channel genes. Using this approach, we could evaluate associations between the responsiveness to ASMs and all coding SNPs, which are more likely to have a functional impact.

## Methods

### Study design

We retrospectively reviewed the medical records of all patients treated and followed up for epilepsy at the Neurological Department of Kaohsiung Chang Gung Memorial Hospital, Taiwan between January 2010 and December 2018. This study was approved by the Chang Gung Medical Foundation Institutional Review Board (IRB No.: 104-1961C, 104-2308B, 201800433B0D001, and 201901274B0D001).

### Definitions and criteria

The inclusion criteria were patients with epilepsy aged > 20 years who took at least one SCN blocking ASM and followed up for at least 12 months. The SCN blocking ASMs (SCN-ASMs) investigated in this study included phenytoin, carbamazepine, lamotrigine, oxcarbazepine, lacosamide, zonisamide, topiramate, and valproic acid [22]. Since some SCN-ASMs also have broad-spectrum non-SCN mechanisms, such as valproate acid, topiramate, and zonisamide, we further divided the ASMs into a “predominant SCN blocking ASM (predominant SCN-ASM)” subgroup composed of phenytoin, carbamazepine, lamotrigine, oxcarbazepine, and lacosamide [22]. Patients were excluded if they had a history of psychogenic nonepileptic seizures, those who could not provide information about seizure frequency, those who did not take any SCN-ASMs, and those with poor drug compliance. Patients with epilepsy syndromes that involved a known genetic mutation of SCN genes or were known to be refractory to multiple ASMs, such as Dravet syndrome and Lennox Gastaut syndrome, were also excluded [23, 24].

We evaluated the efficacy of the ASMs according to the seizure frequency after adding the last SCN-ASM and categorized the efficacy according to the International League Against Epilepsy (ILAE) consensus [3]. A response to the SCN-ASMs was defined as seizure freedom lasting for ≥12 months after taking the last SCN-ASM. Patients not achieving seizure-free were further divided into partial response and failure to SCN-ASMs. A partial response was defined as a seizure frequency < 50% of the pretreatment seizure frequency during the use of the last SCN-ASM with an adequate trial. Failure to the SCN-ASMs was defined as persistent seizures at > 50% of the pretreatment seizure frequency after the last SCN-ASM with an adequate trial. If the SCN-ASM trial was stopped before the outcome was known or if the data required to assess the outcomes were missing, the response was categorized as being “unknown” [25]. The classifications for the efficacy of the predominant SCN-ASMs were the same as for the SCN-ASMs.

Clinical data including gender, onset age, type of seizure, etiology of seizure, seizure frequency, other medical diseases, the type and maintenance dose of ASM, electroencephalography, and brain imaging were collected. Seizure types and epilepsy syndromes were classified according to the 2017 ILAE classification and terminology [26, 27].

### Targeted next-generation sequencing gene panels and calling of SNPs

Genomic DNA was extracted from peripheral blood leukocytes using QIAGEN DNA extraction kits (Qiagen, Germany), according to the manufacturer’s instructions. A customized panel including four SCN genes (*SCN1A*, *SCN1B*, *SCN2A*, *SCN9A*) was used to sequence all of the coding regions including at least 10 base pairs (bp) flanking sequences of the intron/exon boundaries (primers available upon request). The library was prepared using a multiplex polymerase chain reaction to amplify target regions and then sequenced using an Illumina MiSeq platform with 2 × 300 bp paired-end runs. Raw read data were processed with standard bioinformatics pipelines using a Galaxy platform. Briefly, the reads were mapped to the human reference genome (GRCh37) with BWA-MEM and called using FreeBayes. Variants were annotated with wANNOVAR. Only variants found in dbSNP build 147 were selected for further analysis. The mean read depth of the panel was 300.1x and 78.6% coverage of the target region for at least 20 reads.

### Statistical analysis

The χ2 test or Fisher’s exact test is applied to test if the genetic polymorphisms are in agreement with Hardy-Weinberg equilibrium. Those who did not meet Hardy–Weinberg equilibrium were excluded from further study. The χ2 test or Fisher’s exact test was used where appropriate to assess differences in genotype and allele frequencies between the patients who had a response to ASMs and not achieving seizure-free. Among the patients who did not achieve seizure-free, the same statistical method was applied to assess differences between the patients with a partial response and failure to ASMs. Bonferroni correction was applied to rule out false-positive associations after multiple comparisons, with p < α/n (*n* = total number of statistical tests) considered significant when comparing genotypes and alleles with the drug response.

## Results

### Demographic characteristics

During the study period, 214 Taiwanese patients who took SCN-ASMs were enrolled in the study. One patient was excluded due to noncompliance and 13 patients had an unknown response to SCN-ASMs. The remaining 200 patients were then analyzed.

The clinical characteristics of the patients taking SCN-ASMs are shown in Table 1. One hundred and nine (54.5%) patients were classified as having a response to SCN-ASMs. Focal epilepsy was the most prevalent seizure type (60.5%) followed by generalized (25.5%) and unclassified (14.0%). Most of the patients had an unknown etiology (71.0%). The known etiologies included structural lesions (19.0%), central nervous system (CNS) infections (6.0%), genetic (2.0%), and autoimmune (2.0%). The age at onset was significantly older in the patients with a response to SCN-ASMs. The clinical characteristics of patients not achieving seizure-free to SCN-ASMs (*n* = 91) were shown in Table 2, which were categorized into having partial response and failure.

**Table 1:** Demographic data of patients taking sodium channel blocking antiseizure medications

	All (*n* = 200)	Response (*n* = 109)	Not seizure-free (*n* = 91)	*p*
Onset age (year)	16.0 (8.0–24)	17.5 (10.0–26.0)	12.8 (5.5–20)	0.001
Male	100 (50.0)	54 (49.5)	46 (50.5)	reference
Female	100 (50.0)	55 (50.5)	45 (49.5)	0.887
Seizure type				
Focal	121 (60.5)	62 (56.9)	59 (64.8)	0.265
Generalized	51 (25.5)	24 (22.0)	27 (29.7)	
Unspecified	28 (14.0)	23 (21.1)	5 (5.5)	
Etiology				
Structural	38 (19.0)	17 (15.6)	21 (23.1)	0.760
CNS infection	12 (6.0)	6 (5.5)	6 (6.6)	
Genetic	4 (2.0)	1 (0.9)	3 (3.3)	
Autoimmune	4 (2.0)	1 (0.9)	3 (3.3)	
Unknown	142 (71.0)	84 (77.1)	58 (63.7)	
Number of ASMs				
1	65 (32.5)	58 (53.2)	7 (7.7)	
2	61 (30.5)	37 (33.9)	24 (26.4)	
3	47 (23.5)	12 (11.0)	35 (38.5)	
4	16 (8.0)	2 (1.8)	14 (15.4)	
5	9 (4.5)	0 (0.0)	9 (9.9)	
6	2 (1.0)	0 (0.0)	2 (2.2)	
Concurrent ASMs				
Carbamazepine	49	26	23	
Clobazam	11	2	9	
Gabapentin	2	0	2	
Lacosamide	3	0	3	
Lamotrigine	70	35	35	
Levetiracetam	80	26	54	
Oxcarbazepine	22	7	15	
Perampanel	11	0	11	
Phenobarbital	19	1	12	
Phenytoin	40	22	18	
Pregabalin	3	0	3	
Topiramate	41	13	28	
Valproic acid	72	40	32	
Vigabatrin	4	0	4	
Zonisamide	28	4	24	

Continuous variables were presented as median (interquartile range)

Categorical variables were presented as n (%)

*Abbreviations*: *CNS* central nervous system, *ASM* antiseizure medication

**Table 2:** Demographic data of the patients with partial response and failure to sodium channel blocking antiseizure medications

	All (*n* = 91)	Partial response (*n* = 23)	Failure (*n* = 68)	*p*
Onset age (year)	12.8 (4.5–20.0)	11.0 (3.0–19.5)	13 (6.0–20.0)	0.496
Male	46 (50.5)	11 (47.8)	35 (51.5)	reference
Female	45 (49.5)	12 (52.2)	33 (48.5)	0.476
Seizure type				
Focal	59 (64.8)	14 (60.9)	45 (66.2)	0.811
Generalized	27 (29.7)	8 (34.8)	19 (27.9)	
Unspecified	5 (5.5)	1 (4.3)	4 (5.9)	
Etiology				
Structural	21 (23.1)	5 (21.7)	16 (23.5)	0.659
CNS infection	6 (6.6)	2 (8.7)	4 (5.9)	
Genetic	3 (3.3)	0 (0.0)	3 (4.4)	
Autoimmune	3 (3.3)	0 0.0)	3 (4.4)	
Unknown	58 (63.7)	16 (69.6)	42 (61.8)	
Number of ASMs				
1	7 (7.7)	1 (4.3)	6 (8.8)	
2	24 (26.4)	6 (26.1)	18 (26.5)	
3	35 (38.5)	10 (43.5)	25 (36.8)	
4	14 (15.4)	5 (21.7)	9 (13.2)	
5	9 (9.9)	1 (4.3)	8 (11.8)	
6	2 (2.2)	0 (0.0)	2 (2.9)	
Concurrent ASMs				
Carbamazepine	23	5	18	
Clobazam	9	1	8	
Gabapentin	2	0	2	
Lacosamide	3	1	2	
Lamotrigine	35	9	26	
Levetiracetam	54	13	41	
Oxcarbazepine	15	7	8	
Perampanel	11	1	10	
Phenobarbital	12	2	10	
Phenytoin	18	4	14	
Pregabalin	3	0	3	
Topiramate	28	11	17	
Valproic acid	32	8	24	
Vigabatrin	4	1	3	
Zonisamide	24	5	19	

Continuous variables were presented as median (interquartile range)

Categorical variables were presented as n (%)

*Abbreviations: CNS* central nervous system, *ASM* antiseizure medication

After excluding patients using broad-spectrum mechanism ASMs (valproate acid, topiramate, and zonisamide), 82 patients were classified into the predominant SCN-ASMs subgroup. The clinical characteristics of these 82 patients are presented in Table 3. Among these patients, 58 (70.7%) were responsive to predominant SCN-ASMs. The focal seizure was the most prevalent seizure type (62.2%) followed by generalized (20.7%) and unclassified (17.1%). With regards to the etiology, 15.9% had structural lesions, 4.9% had CNS infections, 1.2% had a genetic etiology, 2.4% had an autoimmune etiology, and 75.6% were unknown. In this group, the age at onset was significantly younger in the patients who responded to predominant SCN-ASMs. The clinical characteristics of patients not achieving seizure-free to predominant SCN-ASMs (*n* = 24) were shown in Table 4, which were categorized into having partial response and failure.

**Table 3:** Demographic data of patients taking predominant sodium channel blocking antiseizure medications

	All (*n* = 82)	Response (*n* = 58)	Not seizure-free (*n* = 24)	*p*
Onset age (year)	17.5 (12.8–26.0)	15.0 (8.0–19.0)	19.0 (15.0–28.0)	0.020
Male	34 (41.5)	24 (41.4)	10 (41.7)	reference
Female	48 (58.5)	34 (58.6)	14 (58.3)	0.981
Seizure type				
Focal	51 (62.2)	34 (58.6)	17 (70.8)	0.382
Generalized	17 (20.7)	12 (20.7)	5 (20.8)	
Unspecified	14 (17.1)	12 (20.7)	2 (8.3)	
Etiology				
Structural	13 (15.9)	8 (13.8)	5 (20.8)	0.072
CNS infection	4 (4.9)	3 (5.2)	1 (4.2)	
Genetic	1 (1.2)	0 (0.0)	1 (4.2)	
Autoimmune	2 (2.4)	0 (0.0)	2 (8.3)	
Unknown	62 (75.6)	47 (81.0)	15 (62.5)	
Number of ASMs				
1	46 (56.1)	40 (69.0)	6 (25.0)	
2	23 (28.0)	13 (22.4)	10 (41.7)	
3	12 (14.6)	5 (8.6)	7 (29.2)	
4	0 (0.0)	0 (0.0)	0 (0.0)	
5	1 (1.2)	0 (0.0)	1 (4.2)	
Concurrent ASMs				
Carbamazepine	29	21	8	
Clobazam	3	2	1	
Gabapentin	1	0	1	
Lacosamide	2	0	2	
Lamotrigine	34	24	10	
Levetiracetam	24	10	14	
Oxcarbazepine	10	6	4	
Perampanel	1	0	1	
Phenobarbital	1	0	1	
Phenytoin	27	18	9	
Vigabatrin	1	0	1	

Continuous variables were presented as median (interquartile range)

Categorical variables were presented as n (%)

*Abbreviations: CNS* central nervous system, *ASM* antiseizure medication

**Table 4:** Demographic data of patients with partial response and failure to predominant sodium channel blocking antiseizure medications

	All (*n* = 24)	Partial response (*n* = 5)	Failure (*n* = 19)	*p*
Onset age	15.0 (6.0–19.5)	6.5 (1.3–14.0)	16.0 (10.0–21.0)	0.586
Male	10 (41.7)	2 (40.0)	8 (42.1)	reference
Female	14 (58.3)	3 (60.0)	11 (57.9)	1.000
Seizure type				
Focal	17 (70.8)	2 (40.0)	15 (78.9)	0.224
Generalized	5 (20.8)	2 (40.0)	3 (15.8)	
Unspecified	2 (8.3)	1 (20.0)	1 (5.3)	
Etiology				
Structural	5 (20.8)	1 (20.0)	4 (21.1)	0.331
CNS infection	1 (4.2)	1 (20.0)	0 (0.0)	
Genetic	1 (4.2)	0 (0.0)	1 (5.3)	
Autoimmune	2 (8.3)	0 (0.0)	2 (10.5)	
Unknown	15 (62.5)	3 (60.0)	12 (63.2)	
Number of ASMs				
1	6 (25.0)	1 (20.0)	5 (26.3)	0.965
2	10 (41.7)	2 (40.0)	8 (42.1)	
3	7 (29.2)	2 (40.0)	5 (26.3)	
4	0 (0.0)	0 (0.0)	0 (0.0)	
5	1 (5.9)	0 (0.0)	1 (5.3)	
Concurrent ASMs				
Carbamazepine	8	2	6	
Clobazam	1	0	1	
Gabapentin	1	0	1	
Lacosamide	2	1	1	
Lamotrigine	10	1	9	
Levetiracetam	14	3	11	
Oxcarbazepine	4	2	2	
Perampanel	1	0	1	
Phenobarbital	1	0	1	
Phenytoin	9	2	7	
Vigabatrin	1	0	1	

Continuous variables were presented as median (interquartile range)

Categorical variables were presented as n (%)

*Abbreviations*: CNS central nervous system, *ASM* antiseizure medication

### Associations between SCN gene SNPs and ASM responsiveness

Using the gene panel, we detected three SNPs in *SCN1A* (rs121918808, rs200176684, and rs201985242), seven in *SCN1B* (rs55742440, rs3746255, rs67486287, rs67701503, rs2305748, rs369032304, and rs72558026), six in *SCN2A* (rs2060198, rs17183814, rs138497939, rs185590667, rs199925238, and rs186154973), and five in *SCN9A* (rs9646771, rs3750904, rs199756028, rs200613417, and rs200956485). With 21 SNPs analyzed, the overall significance level is set to be 0.0024 or less after applying the Bonferroni correction.

Table 5 lists the characteristic of the SNPs from *SCN1A, SCN1B, SCN2A,* and *SCN9A* genes in 200 patients taking SCN-ASMs. Only allele C of rs55742440 in *SCN1B* (OR: 0.466; 95% CI: 0.287–0.754; *p* = 0.0016) was statistically significantly associated with not achieving seizure-free with SCN-ASMs. We further examined whether specific SCN-ASM having a higher risk of not achieving seizure-free, Fisher’s exact test was performed on patients with rs55742440T > C, to find which last SCN-ASM is related to not achieving seizure-free and Bonferroni correction was applied after multiple comparisons. With a total of eight SCN-ASMs and the corrected *p*-value was set at 0.00625 after Bonferroni correction, no particular ASM was significantly associated with not achieving seizure-free (Supplement Table).

**Table 5:** Allele and genotype distribution of patients with response and not achieving seizure free to sodium channel blocking antiseizure medications

Gene	SNPs	Genotypes								Alleles		
			Response (*n* = 109)			Not seizure-free (*n* = 91)				Response A:B; not seizure-free A:B	Odds ratio (95% CI)	P*
		A/B	AA	AB	BB	AA	AB	BB	*p*			
*SCN1A*	rs121918808	C/T	108	1	0	90	1	0	1.0000	217:1; 181:1	0.834 (0.052–13.430)	1.0000
	rs200176684	C/T	108	0	1	91	0	0	1.0000	216:2; 182:0		0.5028
	rs201985242	G/C	109	0	0	90	1	0	0.7017	218:0; 181:1		0.4550
*SCN1B*	rs55742440	T/C	77	29	3	47	35	9	0.0097	183:35; 129:53	0.466 (0.287–0.754)	0.0016
	rs3746255	C/T	104	4	1	85	6	0	0.5169	212:6; 176:6	0.830 (0.263–2.620)	0.7773
	rs67486287	G/C	80	27	2	57	28	6	0.1140	187:31; 142:40	0.589 (0.351–0.987)	0.0431
	rs67701503	C/A	80	27	2	57	28	6	0.1140	187:31; 142:40	0.589 (0.351–0.987)	0.0431
	rs2305748	C/T	80	27	2	57	28	6	0.1140	187:31; 142:40	0.589 (0.351–0.987)	0.0431
	rs369032304	G/A	109	0	0	90	1	0	0.4550	218:0; 181:1		0.4550
	rs72558026	G/A	108	1	0	86	5	0	0.0941	217:1; 177:5	0.163 (0.019–1.409)	0.0965
*SCN2A*	rs2060198	T/A	103	5	1	87	3	1	0.8656	211:7; 177:5	1.174 (0.366–3.765)	0.7913
	rs17183814	G/A	76	30	3	62	27	2	0.9169	182:36; 151:31	0.964 (0.569–1.631)	0.8875
	rs138497939	A/C	107	2	0	91	0	0	0.5016	216:2; 182:0		0.5028
	rs185590667	T/C	108	1	0	91	0	0	1.0000	217:1; 182:0		1.0000
	rs199925238	G/A	108	1	0	91	0	0	1.0000	217:1; 182:0		1.0000
	rs186154973	G/A	108	1	0	91	0	0	1.0000	217:1; 182:0		1.0000
*SCN9A*	rs9646771	T/C	108	1	0	91	0	0	1.0000	217:1; 182:0		1.0000
	rs3750904	T/C	78	26	5	69	20	2	0.6430	182:36; 158:24	1.302 (0.745–2.277)	0.3537
	rs199756028	G/A	108	1	0	91	0	0	1.0000	217:1; 182:0		1.0000
	rs200613417	A/T	109	0	0	90	1	0	0.4550	218:0; 181:1		0.4550
	rs200956485	G/A	108	1	0	91	0	0	1.0000	217:1; 182:0		1.0000

*Abbreviations*: *CI* confidence interval, *SNP* single nucleotide polymorphism

* The significance level is set to be 0.0024 or less using Bonferroni correction

Table 6 lists the associations between the SNPs and the 91 patients with a partial response or failure to SCN-ASMs, none were found to have significant associations. Table 7 lists the associations between the SNPs and the response of 82 patients taking predominant SCN-ASMs and Table 8 lists the associations between the SNPs and the 24 patients with partial response or failure to predominant SCN-ASMs. No SNPs were found to be associated with the drug responsiveness for patients taking predominant SCN-ASMs.

**Table 6:** Allele and genotype distribution of the patients with partial response and failure to sodium channel blocking antiseizure medications

Gene	SNPs	Genotypes								Alleles		
			Partial response (*n* = 23)			Failure (*n* = 68)				Partial response A:B; failure A:B	Odds ratio (95% CI)	P*
		A/B	AA	AB	BB	AA	AB	BB	*p*			
*SCN1A*	rs121918808	C/T	22	1	0	68	0	0	0.2527	45:1; 136:0		0.2527
	rs200176684	C/T	23	0	0	68	0	0	1.0000	46:0; 136:0		1.0000
	rs201985242	G/C	23	0	0	67	1	0	1.0000	46:0; 135:1		1.0000
*SCN1B*	rs55742440	T/C	12	9	2	35	26	7	0.9753	33:13; 96:40	0.946 (0.451–1.982)	0.8875
	rs3746255	C/T	21	2	0	64	4	0	0.6403	44:2; 132:4	1.500 (0.266–8.473)	0.4743
	rs67486287	G/C	13	9	1	44	19	5	0.6175	35:11; 107:29	1.160 (0.525–2.560)	0.7184
	rs67701503	C/A	13	9	1	44	19	5	0.6175	35:11; 107:29	1.160 (0.525–2.560)	0.7184
	rs2305748	C/T	13	9	1	44	19	5	0.6175	35:11; 107:29	1.160 (0.525–2.560)	0.7184
	rs369032304	G/A	23	0	0	67	1	0	1.0000	46:0; 135:1		1.0000
	rs72558026	G/A	22	1	0	64	4	0	1.0000	45:1; 132:4	0.733 (0.080–6.734)	1.0000
*SCN2A*	rs2060198	T/A	23	0	0	64	3	1	0.6765	46:0; 131:5		0.3321
	rs17183814	G/A	17	6	0	45	21	2	0.8849	40:6; 111:25	0.666 (0.255–1.742)	0.4061
	rs138497939	A/C	23	0	0	68	0	0	1.0000	46:0; 136:0		1.0000
	rs185590667	T/C	23	0	0	68	0	0	1.0000	46:0; 136:0		1.0000
	rs199925238	G/A	21	2	0	63	5	0	1.0000	44:2; 131:5		1.0000
	rs186154973	G/A	23	0	0	68	0	0	1.0000	46:0; 136:0		1.0000
*SCN9A*	rs9646771	T/C	13	7	3	48	12	8	0.3272	33:13; 108:28	1.520 (0.707–3.264)	0.2814
	rs3750904	T/C	18	5	0	51	15	2	1.0000	41:5; 117:19	0.751 (0.263–2.140)	0.5902
	rs199756028	G/A	23	0	0	68	0	0	1.0000	46:0; 136:0		1.0000
	rs200613417	A/T	23	0	0	67	1	0	1.0000	46:0; 135:1		1.0000
	rs200956485	G/A	23	0	0	68	0	0	1.0000	46:0; 136:0		1.0000

*Abbreviations*: *CI* confidence interval, *SNP* single nucleotide polymorphism

* The significance level is set to be 0.0024 or less using Bonferroni correction

**Table 7:** Allele and genotype distribution of patients with response and not achieving seizure free to predominant sodium channel blocking antiseizure medications

Gene	SNPs	Genotypes								Alleles		
			Response (*n* = 58)			Not seizure-free (*n* = 24)				Response A:B; not seizure-free A:B	Odds ratio (95% CI)	P*
		A/B	AA	AB	BB	AA	AB	BB	*p*			
*SCN1A*	rs121918808	C/T	57	1	0	24	0	0	1.0000	115:1; 48:0		1.0000
	rs200176684	C/T	57	0	1	24	0	0	1.0000	114:2; 48:0		0.5834
	rs201985242	G/C	58	0	0	24	0	0	1.0000	116:0; 48:0		1.0000
*SCN1B*	rs55742440	T/C	40	17	1	12	8	4	0.0310	97:19; 32:16	0.392 (0.180–0.851)	0.0159
	rs3746255	C/T	57	1	0	22	2	0	0.2036	115:1; 46:2	0.2 (0.017–2.260)	0.2052
	rs67486287	G/C	41	16	1	17	5	2	0.3292	98:18; 39:9	0.796 (0.330–1.923)	0.6101
	rs67701503	C/A	41	16	1	17	5	2	0.3292	98:18; 39:9	0.796 (0.330–1.923)	0.6101
	rs2305748	C/T	41	16	1	17	5	2	0.3292	98:18; 39:9	0.796 (0.330–1.923)	0.6101
	rs369032304	G/A	58	0	0	24	0	0	1.0000	116:0; 48:0		1.0000
	rs72558026	G/A	57	1	0	20	4	0	0.0241	115:1; 44:4	0.096 (0.010–0.880)	0.0261
*SCN2A*	rs2060198	T/A	54	4	0	24	0	0	0.3158	112:4; 48:0		0.3222
	rs17183814	G/A	41	14	3	14	9	1	0.4452	96:20; 37:11	0.701 (0.306–1.603)	0.5110
	rs138497939	A/C	57	1	0	24	0	0	1.0000	115:1; 48:0		1.0000
	rs185590667	T/C	57	1	0	24	0	0	1.0000	115:1; 48:0		1.0000
	rs199925238	G/A	54	4	0	21	3	0	0.4418	112:4; 45:3	0.536 (0.115–2.490)	0.6746
	rs186154973	G/A	57	1	0	24	0	0	1.0000	115:1; 48:0		1.0000
*SCN9A*	rs9646771	T/C	39	12	7	14	5	5	0.5625	90:26; 33:15	0.636 (0.300–1.346)	0.2351
	rs3750904	T/C	44	11	3	18	6	0	0.6353	99:17; 42:6	1.202 (0.433–3.261)	0.7184
	rs199756028	G/A	57	1	0	24	0	0	1.0000	115:1; 48:0		1.0000
	rs200613417	A/T	58	0	0	23	1	0	0.2926	116:0; 47:1		0.2927
	rs200956485	G/A	57	1	0	24	0	0	1.0000	115:1; 48:0		1.0000

*Abbreviations*: *CI* confidence interval, *SNP* single nucleotide polymorphism

* The significance level is set to be 0.0024 or less using Bonferroni correction

**Table 8:** Allele and genotype distribution of patients with partial response and failure to predominant sodium channel blocking antiseizure medications

Gene	SNPs	Genotypes								Alleles		
			Partial response (*n* = 5)			Failure (*n* = 19)				Partial response A:B; failure A:B	Odds ratio (95% CI)	p*
		A/B	AA	AB	BB	AA	AB	BB	*p*			
*SCN1A*	rs121918808	C/T	5	0	0	19	0	0	1.0000	10:0; 38:0		1.0000
	rs200176684	C/T	5	0	0	19	0	0	1.0000	10:0; 38:0		1.0000
	rs201985242	G/C	5	0	0	19	0	0	1.0000	10:0; 38:0		1.0000
*SCN1B*	rs55742440	T/C	2	2	1	10	6	3	1.0000	6:4; 26:12	1.444 (0.342–6.086)	0.7118
	rs3746255	C/T	5	0	0	17	2	0	1.0000	10:0; 36:2		1.0000
	rs67486287	G/C	2	3	0	15	2	2	0.0785	7:3; 32:6	2.286 (0.457–11.426)	0.3698
	rs67701503	C/A	2	3	0	15	2	2	0.0785	7:3; 32:6	2.286 (0.457–11.426)	0.3698
	rs2305748	C/T	2	3	0	15	2	2	0.0785	7:3; 32:6	2.286 (0.457–11.426)	0.3698
	rs369032304	G/A	5	0	0	19	0	0	1.0000	10:0; 38:0		1.0000
	rs72558026	G/A	5	0	0	15	4	0	0.5440	10:0; 34:4		0.5665
*SCN2A*	rs2060198	T/A	5	0	0	19	0	0	1.0000	10:0; 38:0		1.0000
	rs17183814	G/A	2	3	0	12	6	1	0.4797	7:3; 30:8	1.607 (0.337–7.658)	0.6754
	rs138497939	A/C	5	0	0	19	0	0	1.0000	10:0; 38:0		1.0000
	rs185590667	T/C	5	0	0	19	0	0	1.0000	10:0; 38:0		1.0000
	rs199925238	G/A	4	1	0	17	2	0	0.5212	9:1; 36:2	2.000 (0.163–24.590)	1.0000
	rs186154973	G/A	5	0	0	19	0	0	1.0000	10:0; 38:0		1.0000
*SCN9A*	rs9646771	T/C	0	3	2	14	2	3	0.0092	3:7; 30:8	8.75 (1.836–41.693)	0.0059
	rs3750904	T/C	4	1	0	14	5	0	1.0000	9:1; 33:5	0.733 (0.076–7.099)	1.0000
	rs199756028	G/A	5	0	0	19	0	0	1.0000	10:0; 38:0		1.0000
	rs200613417	A/T	5	0	0	18	1	0	1.0000	10:0; 37:1		1.0000
	rs200956485	G/A	5	0	0	19	0	0	1.0000	10:0; 38:0		1.0000

*Abbreviations*: *CI* confidence interval, *SNP* single nucleotide polymorphism

* The significance level is set to be 0.0024 or less using Bonferroni correction

## Discussion

In this study, the C allele of rs55742440 in *SCN1B* was statistically significantly associated with not achieving seizure-free with SCN-ASMs among Taiwanese patients. This finding is consistent with the new perspective that the beta subunit of SCN, which is encoded by *SCN1B* [28], may influence the function of SCNs.

Voltage-gated SCNs are composed of alpha and beta subunits [29]. The alpha subunits form the functional pore of the channel, and the beta subunits serve to modulate the channel’s biophysical properties [30]. Mutations of the alpha subunits have been shown to cause Dravet syndrome and generalized epilepsy with febrile seizures plus [10], which could also be caused by mutations in the beta subunits [10, 31, 32]. No previous study has reported an association between *SCN1B* and ASM responsiveness in humans, although animal models have shown that mutations of SCN1B can reduce sensitivity to ASMs [33–35]. Our findings suggest that SNPs in *SCN1B* could cause functional alterations of SCNs, thereby affecting the responsiveness to ASMs.

In the current study, rs55742440T > C in *SCN1B* was associated with not achieving seizure-free with SCN-ASMs. The rs55742440T > C SNP causes a missense change of the amino acid (p.Leu210Pro) located in the intracellular C-terminal domain of *SCN1B*. According to the gnomAD database [36], it is prevalent across all ethnicities (MAF = 0.38), not only in the East Asian population (MAF = 0.27) where this study cohort origin. Therefore, this SNP is unlikely to be pathogenic for epilepsy based on the ACMG guideline. Nevertheless, the variant has been reported in patients with Brugada syndrome and arrhythmogenic right ventricular cardiomyopathy [37–39], it was also demonstrated to reduce sodium channel current in cardiomyocytes together with another missense variant [39]. It is possible that the SCN1B variant modifies the function of alpha subunits of voltage-gated sodium channels and makes it less responsive to SCN-ASMs via a currently unknown mechanism. Taken together, rs55742440 may affect sodium channel function in the heart and brain as well as responsiveness to medications targeting sodium channels. Further functional investigations are warranted to elucidate this issue.

When the analysis was done for the more restricted predominant SCN-ASMs group, no association of drug responsiveness was found for rs55742440T > C in *SCN1B*. This could have resulted from the decrease in patient numbers in the predominant SCN-ASMs group. We also observed a difference in the age at onset between the responsiveness to SCN-ASMs and the more restricted predominant SCN-ASMs. In previous studies, the age at onset was not related to the responsiveness to ASMs [2, 40–42]. This could be caused by bias due to the relatively small sample size in our study, which is crucial to genetic association studies [43].

Our study was limited by the relatively small sample size and the need for multiple statistical tests for association studies. The sample size is a crucial component of the power to detect a causal variant in the genetic association study [43]. In practice, however, the sample size was limited by the amount of well-characterized clinical samples and the costs of sequencing. With more delicate sample characterizing, the less sample size one got. As in our case, the sample size dropped as we changed the response of the ASM to a more restricted “predominant SCN-ASMs”, which resulted in a loss of power and failure to detect any significant signals. A potential solution to increase the sample size would be performing a meta-analysis [21, 44] or international research collaboration, such as the EpiPGX consortium [45]. Although the costs of sequencing are dropping due to recent advancements in genetic sequencing technology, the price is still too expensive for many researchers. Even with affordable prices, the advanced sequencing technique generates an even greater amount of data. More genetic data would accompany multiple statistical tests that could increase false-positive results [46]. To counter this problem, multiple test correction was applied and the traditional Bonferroni correction may be overly conservative that may not be able to find a significant association [47]. New statistical methods for genetic association studies have been developed [48] and, in the future, we might have better tools to process these immense amounts of data to gain a better insight into the association between genotypes and clinical phenotypes.

We only focus on the variation of SCN genes, but other mechanisms may also contribute to drug resistance epilepsy, including drug transporters and proteins involved in the metabolism of ASMs. The efflux transporters at the endothelial cells of the blood-brain barrier (BBB) may hamper the ASMs’ ability to enter the central nervous system and decrease their concentration at the epileptogenic tissues [4]. ATP binding cassette (ABC) transporter superfamily is the major efflux transporter on the BBB that may limit the access of ASMs to the brain [49] but various studies had conflicting results about the association of genetic polymorphism of ABC transporter genes and the responsiveness of ASM [50–52]. The solute carrier (SLC) protein is another transporter protein that is responsible for drug transport in the brain and its polymorphisms have functional significance in terms of the pharmacokinetics of many drugs [53]. Currently, no studies focus on the association of the polymorphism of SLC proteins and the responsiveness of ASMs. Drug-metabolizing genes are polymorphic and can influence the biotransformation of many drugs [54]. Enzyme variants may alter the responsiveness of the ASMs, which makes them ineffective. One Taiwanese study revealed the polymorphism of EPHX was related to a higher dose of carbamazepine for seizure control [55]. Another Chinese study showed the polymorphism of UGT was associated with a higher dose of oxcarbazepine in controlling seizures [56]. The magnitude of the factors influencing the responsiveness of ASM is great, a larger population study and newer methods will be needed to explore this complex interaction.

The response of an ASM is currently unpredictable and usually requires trial at the cost of ongoing seizures or the occurrence of adverse reactions. The pursuit of predicting the responsiveness to ASMs using genetic information, such as SNPs, has not been very successful [57, 58], and conflicting results have been observed in gene association studies [12, 13, 15, 16, 18, 59, 60]. The recent development of polygenic risk scores could be a promising method to unravel the complex relationship between genetic background and drug responsiveness [61, 62]. A recent large whole-exome sequencing study found that rare coding damaging variants were marginally enriched in genes involved in the pharmacokinetics of valproic acid in patients resistant to valproic acid [25]. This is similar to the findings in the present study, in that coding variants of SCN genes (particularly *SCN1B*) affected the responsiveness to SCN-ASMs. The predictive ability of these findings still needs to be replicated in different study cohorts.

## Conclusion

We identified rs55742440T > C in *SCN1B* was associated with not achieving seizure-free with SCN-ASMs. although the beta subunits of the SCN were considered as an auxiliary component, our finding supports the concept that the beta subunit could influence the function of the SCN. Based on this finding, further function test about the beta subunit and this SNP is needed to explore the role of rs55742440 in *SCN1B.*

## Supplementary Information

**Additional file 1: Supplement Table.** The response of individual sodium channel blocking antiseizure medications in patients with rs55742440T > C.

## Data Availability

The datasets generated and analysed during the current study cannot be made openly due to ethical concern but are available from the corresponding author on reasonable request.

## Abbreviations

ASM: Antiseizure medication
SCN: Sodium channel
SNP: Single-nucleotide polymorphisms
