# Supplementary material for: Response to Sodium Channel blocking Antiseizure medications and coding polymorphisms of Sodium Channel genes in Taiwanese epilepsy patients
Source: BMC Neurol. 2021 Sep 23;21:367. doi: 10.1186/s12883-021-02395-2 (PMC8459515; doi:10.1186/s12883-021-02395-2)
Supplement: Supplementary file 1 — Additional file 1: Supplement Table. The response of individual sodium channel blocking antiseizure medications in patients with rs55742440T > C. [file 12883_2021_2395_MOESM1_ESM.docx]

**Response to Sodium Channel Blocking Antiseizure Medications and Coding Polymorphisms of Sodium Channel Genes in Taiwanese Epilepsy Patients**

Chih-Hsiang Lin^1^, Chen-Jui Ho^1^, Yan-Ting Lu^1^, Meng-Han Tsai^1,2^*

^1^Department of Neurology, Kaohsiung Chang Gung Memorial Hospital, Colleague of Medicine, Chang Gung University, Kaohsiung, Kaohsiung City 83301, Taiwan.

^2^School of Medicine, College of Medicine, Chang Gung University, Taoyuan, Taiwan

*Corresponding to:

Prof Meng-Han Tsai, MD, PhD

Department of Neurology, Kaohsiung Chang Gung Memorial Hospital, College of Medicine, Chang Gung University, Kaohsiung City 83301, Taiwan

Email address: [menghan@cgmh.org.tw](mailto:menghan@cgmh.org.tw) (M-H Tsai)

Supplement Table. The response of individual sodium channel blocking antiseizure medications in patients with rs55742440T>C.

| Last ASM | Response (n=32) | Not seizure free (n=44) | Odds ratio (95% CI) | *p** |
| --- | --- | --- | --- | --- |
| Carbamazepine | 10 | 5 | 3.546 (1.074-11.701) | 0.042 |
| Lacosamide | 0 | 1 |  | 1.000 |
| Lamotrigine | 6 | 5 | 1.800 (0.497-6.516) | 0.511 |
| Oxcarbazepine | 1 | 6 | 0.204 (0.023-1.789) | 0.228 |
| Phenytoin | 2 | 2 | 1.400 (0.187-10.503) | 1.000 |
| Topiramate | 3 | 9 | 0.402 (0.100-1.625) | 0.222 |
| Valproic acid | 9 | 4 | 3.913 (1.083-14.139 | 0.0362 |
| Zonisamide | 1 | 12 | 0.086 (0.011-0.702) | 0.0112 |

Abbreviations: ASM= antiseizure medication; CI=confidence interval.

* The significance level is set to be 0.00625 or less using Bonferroni correction.
